# Outcomes of Simultaneous Liver-Kidney Transplant and Kidney After Liver Transplant Using the Safety Net Criteria—A Single-center Experience

**DOI:** 10.1097/TXD.0000000000001940

**Published:** 2026-04-22

**Authors:** Srijan Tandukar, Vishnu S. Potluri, Peter Abt, David E. Kaplan, Therese Bittermann, Behdad Besharatian, Maarouf A. Hoteit, Matthew Levine, Arwin Thomasson, Sarah Hryzak, Anjana Murali, Shreeya Thapaliya, Karthik Ranganna, Roy D. Bloom, Ty B. Dunn, Mary Ann Lim

**Affiliations:** 1 Renal Electrolyte and Hypertension Division, Department of Medicine, University of Pennsylvania, Philadelphia, PA.; 2 Division of Transplant Surgery, Department of Surgery, University of Pennsylvania, Philadelphia, PA.; 3 Division of Gastroenterology and Hepatology, University of Pennsylvania, Philadelphia, PA.; 4 Department of Nephrology, RCSI Hospital Group, Dublin, Ireland.; 5 Penn Transplant Institute, University of Pennsylvania, Philadelphia, PA.; 6 Nephrology Associates of North East Jacksonville, Jacksonville, FL.; 7 Department of Medicine, University of Pennsylvania, Philadelphia, PA.; 8 Division of Transplant Surgery, Department of Surgery, Medical College of Wisconsin, Milwaukee, WI.

## Abstract

**Background.:**

The impact of revised Organ Procurement and Transplantation Network policy defining eligibility for simultaneous liver-kidney (SLK) and the safety net criteria for kidney after liver (KAL) transplantation remains insufficiently characterized.

**Methods.:**

We conducted a retrospective study of adults (>18 y) evaluated for liver transplant alone (LTA), SLK, or KAL at the University of Pennsylvania from August 10, 2017, to February 28, 2023. The primary outcome was mortality among SLK and KAL recipients and those patients waitlisted for KAL. Secondary outcomes included native kidney recovery in LTA recipients, estimated glomerular filtration rate (eGFR) at 1 y post-kidney transplant, and time between liver and kidney transplants.

**Results.:**

Of 1655 patients evaluated, 57 (3.4%) met SLK criteria; 49 (86%) underwent SLK and 8 (14%) received LTA. Among 1598 LTA candidates, 1010 (63%) were waitlisted and 717 (71%) received LTA. After excluding 9 early deaths (1.3%) that were unrelated to KAL delay, 67 survivors (9.5%) met KAL safety net criteria. Thirty-four (50.8%) were waitlisted (15 transplanted over a median of 220 d), 30 (44.8%) declined, and 3 (4.8%) remained under evaluation. Mortality was 4.1% after SLK and no deaths occurred after KAL >3.7 and 3.1 y of follow-up, with 3 deaths (8.8%) among KAL waitlisted patients. Of the 16 KAL waitlisted patients alive without kidney transplant at last follow-up, 2 (12.5%) were delisted after documented renal recovery and an additional 9 (56.3%) had eGFR >20 ml/min/1.73 m^2^ and were being considered for delisting; together, 11 (68.8%) met predefined criteria for substantial native kidney recovery. One-year median eGFR was similar (SLK 51 versus KAL 54 mL/min/1.73 m^2^; *P* = 0.6).

**Conclusions.:**

Early post-LTA mortality was unrelated to delayed KAL transplantation. Recovery of native kidney function while waiting for KAL was frequent. KAL transplants occurred within a year of LTA with favorable survival and graft outcomes.

## INTRODUCTION

Kidney disease has been associated with increased morbidity and mortality of liver transplant candidates and recipients, leading to the addition of serum creatinine as a variable in the Model for End-Stage Liver Disease (MELD) score, and the utilization of simultaneous liver and kidney (SLK) transplants.^1-9^ SLK transplants have shown improved survival in patients with true dual organ failure since its first introduction in 1983; this led to tripling of SLK transplants since then, with wide practice variation across centers, raising concerns about equity and organ utilization.^10-14^ A new SLK allocation policy introduced in 2017, intended to: (1) standardize medical eligibility criteria for SLK transplants, (2) minimize the prioritization of kidney allocation to liver candidates over highly sensitized kidney candidates, (3) prioritize patients deprived of an SLK transplant (because of stricter eligibility criteria) for kidney after liver (KAL) transplant, and (4) ensure regional and national SLK allocation to be consistent with those that govern deceased donor liver transplants.^15^

Under this policy, candidates may qualify for an SLK transplant via the chronic kidney disease (CKD), sustained acute kidney injury (AKI), or metabolic disease criteria. Since implementation, while the absolute number of SLK transplants increased, the proportion of SLK to deceased donor kidney transplants decreased (from 5.1% in 2017 to 4% in 2022) without impacting short-term outcomes after a 2-y look back.^16,17^ Moreover, data looking at the first 18 mo post-policy change showed that prior liver transplantation did not affect sensitization, and subsequent KAL transplantation through the safety net was accomplished within 180 d in one-third of KAL listed patients.^18^ Registry data lacked details such as causes of death in SLK recipients, outcomes of KAL waitlisted patients, early (<60 d) deaths after liver transplant alone (LTA), and the status of KAL candidates not yet transplanted. Outcomes were also unclear for patients listed for SLK but ultimately receiving LTA, or for those eligible for SLK who were never considered and underwent LTA instead. Additionally, some post-LTA patients meeting estimated glomerular filtration rate (eGFR) <20 mL/min/1.73 m^2^ criteria were declined for KAL under the safety net pathway, potentially introducing selection bias in comparing SLK and KAL recipients, while reasons for such declines remain poorly defined.

In this study, we analyze center-level data to evaluate the impact of policy changes on SLK utilization, KAL listing and transplantation via the safety net, and outcomes under the more stringent SLK criteria. Through detailed chart reviews, we identify causes of death among KAL waitlisted patients, early post-LTA mortality, native kidney recovery while waitlisted, outcomes of SLK-eligible patients managed with LTA, and reasons for KAL declines under the safety net pathway.

## MATERIALS AND METHODS

### Patient Selection and Study Design

We reviewed all patients ≥18 y who were evaluated for a LTA, SLK, or KAL transplant at the University of Pennsylvania between August 10, 2017, and February 28, 2023, as our initial cohort. We identified patients that were eligible for SLK transplant but subsequently underwent an LTA to understand these patient’s clinical course. We also identified those candidates who were declined for LTA or SLK and those who were never evaluated for an SLK transplant despite fulfilling eligibility criteria and instead underwent LTA. We tracked these patients to see if their outcomes were impacted by not having a kidney transplant in addition to a liver transplant and if they went on to require a KAL transplant.

Patients who died within 60 d of LTA were excluded because they were not alive long enough to be considered for a KAL transplant, although we examined their causes of deaths. Among the patients who were still alive at 60 d, we identified patients that were evaluated for KAL transplant and those that were never eligible or referred for a KAL transplant. We examined the clinical course of these patients waitlisted for KAL, including whether they underwent a kidney transplant, were delisted because of native kidney recovery, died, or their final waitlist status at the time of data extraction. Additionally, we also identified patients that were declined for a KAL transplant, the reasons for the decision and their clinical course.

This is a retrospective observational study that was approved by the Institutional Review Board (IRB) of the University of Pennsylvania (IRB protocol number 853034). All pertinent patient data were obtained from manual chart review of the patients from the electronic health records. The study complies with the Declarations of Helsinki and Istanbul. The requirement for written informed consent was waived by the IRB.

### Immunosuppression

Induction for SLK and LTA recipients was typically a methylprednisolone bolus, with occasional use of basiliximab (N = 1) or rabbit antithymocyte globulin (N = 1) in SLK. SLK maintenance was generally tacrolimus, mycophenolate mofetil (1 switched to azathioprine), and prednisone, while LTA patients received tacrolimus, azathioprine, and prednisone (2 on dual therapy without azathioprine). KAL induction included thymoglobulin (N = 7), basiliximab (N = 7), or methylprednisolone alone (N = 1). KAL maintenance was with tacrolimus, mycophenolate mofetil, and prednisone.

### Outcomes

The primary outcome was mortality in SLK and KAL recipients and those on the KAL waitlist before receiving a kidney transplant. For KAL recipients, the day of kidney transplant was the starting point for assessing mortality while for KAL waitlisted patients, the mortality was assessed from the day of the liver transplant. Secondary outcomes were cause of death, native kidney function recovery with subsequent delisting from the KAL waitlist, kidney transplant function in SLK and KAL recipients at 1-y, acute kidney and liver transplant rejection episodes, and time between KAL initial waitlisting and transplant, between KAL waitlist activation and transplant, and between LTA and KAL transplant. We also investigated the clinical course in patients who were approved for SLK transplant and underwent LTA, those that were eligible for SLK transplant but were never evaluated for an SLK transplant and underwent LTA, those that were declined for a KAL or LTA, delisted or died while waiting on the LTA or KAL waitlist, and the cause of deaths in patients within 60 d of LTA.

### Statistical Analysis

Descriptive statistics were used to summarize the findings. Normally distributed continuous data are presented as mean and SD, whereas nonnormally distributed continuous data are presented as median and interquartile range. Continuous data are compared using Student *t* test and Kruskal-Wallis test as appropriate. Categorical data are presented as proportions with comparisons made using chi-square test and Fisher exact test as appropriate.

## RESULTS

### Study Population

Among 1655 patients evaluated for liver transplant, 57 (3.4%) met criteria for SLK transplant via the CKD (N = 54) or metabolic disease (N = 3 with primary hyperoxaluria) pathways; none qualified via the AKI pathway. Of these, 49 (86%) underwent SLK, while 8 (14%) received LTA including 3 approved but not transplanted as SLK, 2 declined, and 3 eligible but not considered.

The remaining 1598 patients (96.6%) not eligible for SLK were evaluated for LTA. Among them, 1010 (63.2%) were waitlisted, 188 (11.8%) were in the evaluation phase needing representation, and 400 (25%) were declined. Of the waitlisted patients, 717 (70.9%) received LTA, 241 (23.9%) remained on the waitlist, and 52 (5.1%) were removed at the time of data collection.

**FIGURE 1.**
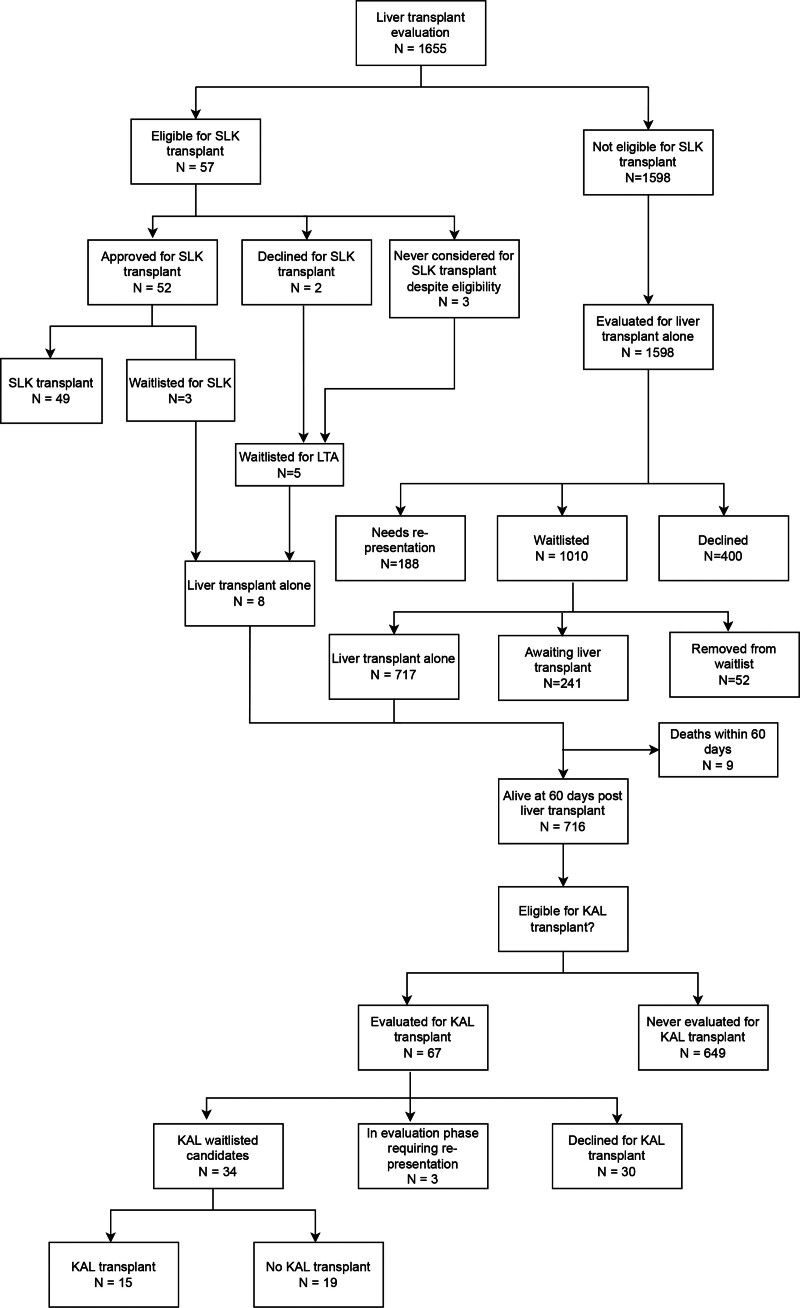
Flowchart showing the number of patients at different steps of SLK, LTA, and KAL transplantation. KAL, kidney after liver; LTA, liver transplant alone; SLK, simultaneous liver-kidney.

Among 725 total LTA recipients, 9 (1.3%) died within 60 d. Of the 716 survivors, 67 (9.5%) met safety net criteria for KAL transplant: 34 (50.7%) were waitlisted (15 transplanted, 19 inactive), 30 (44.8%) were declined, and 3 (4.5%) required representation (Figure 1).

### Recipient and Donor Characteristics

Recipient and donor characteristics are summarized in Table 1 (**Table S1, SDC**, https://links.lww.com/TXD/A852). Compared with SLK recipients, KAL recipients received kidneys from older donors (mean age 41 versus 33 y; *P* = 0.02) with higher terminal creatinine (2.12 versus 0.84 mg/dL; *P* < 0.001) and demonstrated higher sensitization (panel reactive antibody >0: 33.3% versus 4%). Fewer KAL recipients received low Kidney Donor Profile Index (KDPI; 0%–20%) kidneys (6.7% versus 26.5%; *P* = 0.18). One donor kidney (2.04%) was pumped before SLK transplant and none of the kidneys were pumped before KAL transplant.

**TABLE 1.** Baseline characteristics of SLK and KAL transplant recipients and donors

Baseline characteristics	SLK transplant (N = 49)	KAL transplant (N = 15)	*P*
Recipient age	52 (13)	58 (11)	0.12
Recipient sex			0.48
Male	21 (42.9%)	8 (53.3%)	
Recipient ethnicity			0.69
Black	10 (20.4%)	2 (13.3%)	
White	30 (61.2%)	11 (73.3%)	
Others	9 (18.4%)	2 (13.3%)	
Recipient diabetes	21 (42.9%)	4 (26.7%)	0.26
Cause of kidney disease			0.74
Hepatorenal syndrome	15 (30.6%)	6 (40%)	
Hepatorenal syndrome and other possible causes	9 (18.4%)	3 (20%)	
Other causes	25 (51%)	6 (40%)	
Dialysis at the time of listing	21 (42.9%)	11 (73.3%)	0.04
Dialysis at the time of transplant	34 (69.4%)	11 (73.3%)	0.77
eGFR at the time of transplant (for those not on dialysis, mL/min/1.73 m^2^)	25 (12)	15 (1)	0.13
Cause of liver disease			0.05
Alcoholic cirrhosis	6 (12.2%)	6 (40%)	
Hepatocellular carcinoma	6 (12.2%)	2 (13.3%)	
NASH cirrhosis	2 (4.1%)	2 (13.3%)	
Primary biliary cirrhosis	3 (6.1%)	1 (6.7%)	
Others	32 (65.3%)	4 (26.7%)	
Acuity of liver disease			0.001
Acute	0 (0%)	3 (20%)	
Chronic	49 (100%)	12 (80%)	
Panel reactive antibody			0.03
0	47 (96%)	10 (66.7%)	
>0	2 (4%)	5 (33.3%)	
Blood group			0.98
A	19 (38.8%)	6 (40%)	
AB	2 (4.1%)	1 (6.7%)	
B	10 (20.4%)	3 (20%)	
O	18 (36.7%)	5 (33.3%)	
EPTS score			0.11
0%–20%	17 (34.7%)	2 (13.3%)	
20%–100%	32 (65.3%)	13 (86.7%)	
MELD score	32 (5)	33 (7)	0.66
ICU status at time of transplant	8 (16.3%)	0 (0%)	0.1
Kidney replacement modality at the time of transplant			0.54
Continuous renal replacement therapy	3 (6.1%)	0 (0%)	
Intermittent hemodialysis	25 (51.1%)	11 (73.3%)	
Peritoneal dialysis	3 (6.1%)	4 (26.7%)	
KDPI score			0.18
0%–20%	13 (26.5%)	1 (6.7%)	
21%–35%	16 (32.7%)	8 (53.3%)	
36%–85%	20 (40.8%)	6 (40%)	
86%–100%	0 (0%)	0 (0%)	
Donor age	33 (11)	41 (9.8)	0.02
Donor sex			0.2
Male	17 (34.7%)	8 (53.3%)	
Donor ethnicity			0.56
Black	10 (20.4%)	2 (13.3%)	
White	33 (67.3%)	9 (60%)	
Others	6 (12.3%)	4 (26.7%)	
Donor hypertension	5 (10.2%)	3 (20%)	0.31
Donor diabetes	2 (4.1%)	1 (6.7%)	0.79
Donor terminal creatinine	0.84 (0.33)	2.12 (1.99)	<0.001
Donation after circulatory death	1 (2%)	1 (6.7%)	0.37
Hepatitis C serostatus			0.43
Positive	2 (4.1%)	0 (0%)	
HLA mismatch			0.46
3	8 (16.3%)	3 (20%)	
4	15 (30.6%)	2 (13.3%)	
5	17 (34.7%)	8 (53.3%)	
6	9 (18.4%)	3 (13.3%)	
Cold ischemia time, h	12.3 (4.6)	10.7 (3.3)	0.23
Donor/recipient CMV serostatus			0.92
Low risk (–/–)	8 (16.3%)	2 (13.3%)	
Intermediate risk (–/+ or +/+)	24 (49%)	7 (46.7%)	
High risk (+/–)	17 (34.7%)	6 (40%)	
Donor/recipient EBV serostatus			0.35
Low risk (–/–)	1 (2.1%)	0 (0%)	
Intermediate risk (–/+ or +/+)	41 (87.2%)	15 (100%)	
High risk (+/–)	5 (10.6%)	0 (0%)	
Donor kidney pumping	1 (2%)	0 (0%)	1

CMV, cytomegalovirus; EBV, Epstein-Barr virus; eGFR, estimated glomerular filtration rate; EPTS, Expected Posttransplant Survival; ICU, intensive care unit; KAL, kidney after liver; KDPI, Kidney Donor Profile Index; MELD, Model for End-Stage Liver Disease; NASH, nonalcoholic steatohepatitis; SLK, simultaneous liver-kidney.

### Mortality

#### Mortality in SLK and KAL Recipients

There were 2 deaths (4.1%) among SLK recipients over a median of 3.7 y of follow-up: 1 from hepatocholangiocarcinoma around 10 mo posttransplant and a second from complications from graft versus host disease around 8 mo posttransplant (Table 2). Both had normal kidney function and the time of death. There were no deaths among those who received KAL transplants.

**TABLE 2.** Outcomes of simultaneous liver-kidney and kidney after liver transplant

Outcome	SLK transplant (N = 49)	KAL transplant (N = 15)	*P*
Time duration between waitlist activation and transplant, d^*a*^	23 (9–89)	220 (83–374)	0.002
Delayed graft function	18 (36.7%)	5 (33.3%)	0.52
Duration of delayed graft function^*a*^	6 (4–14)	3 (1–8)	0.13
Return to operating room	13 (26.5%)	0 (0%)	0.1
Kidney transplant primary nonfunction	2	0	0.1
Mortality	2	0	0.1
Kidney transplant rejection	1	1	0.15
Liver transplant rejection	9	3	0.69
Kidney transplant graft failure	3	0	0.1
eGFR at 12 mo, mL/min/1.73 m^2^^*a*^	51 (45 – 65)	54 (39 – 59)	0.6

^*a*^Median (Q1–Q3).

eGFR, estimated glomerular filtration rate; KAL, kidney after liver; SLK, simultaneous liver-kidney.

#### Mortality in KAL Waitlisted Candidates

Of the 34 patients who were KAL waitlisted, 3 (8.8%) died while waiting for a KAL transplant: 1 from COVID pneumonia (12-mo post-LTA), 1 from pulseless electrical activity arrest with anoxic brain injury (7-mo post-LTA), and 1 from recurrent cholangiocarcinoma (7-mo post-LTA) over a median of 3.1 y of follow-up.

#### Mortality in LTA Recipients Within 60 d of Transplant

There were 9 deaths (1.3%) among 725 LTA recipients within 60 d. Two patients had primary non function of the liver allograft requiring expedited repeat liver transplants. In 1 patient, the repeat allograft had hepatic necrosis with no flow in the hepatic artery, leading to liver allograft failure and death. The other patient had persistent liver dysfunction in the setting of multiorgan failure with the family opting for comfort care. The cause of deaths in the remaining 8 patients were hemorrhagic shock, cardiogenic shock, unwitnessed fall in the rehab, followed by death shortly thereafter without an exact identifiable cause, cardiac arrest leading to anoxic brain injury and withdrawal of support, acute cerebrovascular accident, and multiorgan failure in the early posttransplant period.

#### Mortality in Patients Evaluated for Liver Transplant

Of 188 patients needing representation before waitlisting, 16 (8.5%) died. Among 1010 waitlisted patients, 55 (22.8%) died while awaiting transplant and 14 (26.9%) died after removal from the waitlist.

### Native Kidney Recovery in KAL Waitlisted Patients

Among 16 waitlisted KAL patients that were still alive without a kidney transplant at the end of follow-up, 2 recovered kidney function and were delisted. The first patient was on dialysis for 24 d before liver transplant. The second patient had AKI presumed from hepatorenal syndrome and had renal recovery to eGFR persistently >35 mL/min/1.73 m^2^ post-LTA and was subsequently removed from the KAL waitlist. Of the remaining 14 patients, 9 (64.3%) had eGFR >20 mL/min/1.73 m^2^ and were being considered for delisting, and 5 (35.7%) were on dialysis with 1 being actively listed for kidney transplant. The reasons for inactivation were: incomplete workup (N = 7), too sick (N = 3), temporarily too well (N = 2), and patient choice (N = 1). Presence of proteinuria or diabetes did not correlate with eGFR <20 mL/min/1.73 m^2^ at last follow-up (60% versus 66.7%; *P* = 0.76 and 50% versus 33.3%; *P* = 0.46) in this small sample size of KAL waitlisted patients.

### Kidney Allograft Function

**FIGURE 2.**
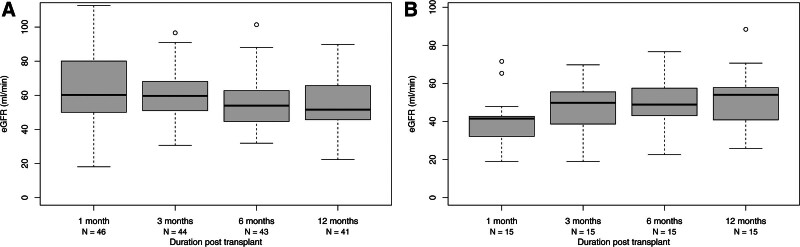
eGFR distribution representing kidney allograft function among recipients within the first year of SLK and KAL transplant. The eGFR for KAL transplant indicate values after the kidney transplant. A, SLK transplant. B, KAL transplant. eGFR, estimated glomerular filtration rate; KAL, kidney after liver; SLK, simultaneous liver-kidney.

The median eGFR in SLK versus KAL cohort at 1-y post-kidney transplant was 51 and 54 mL/min/1.73 m^2^, respectively (*P* = 0.6; Figure 2). Two SLK recipients (4%) and none of the KAL recipients developed primary nonfunction (PNF), comparable to the 5 y before policy change (1 PNF in the SLK group and none in the KAL group).

Biopsy-proven acute tubular necrosis from persistent hypotension was deemed to be the cause of PNF in the first SLK recipient who had positive biliary fluid cultures and atrial fibrillation with rapid ventricular rate posttransplant. In the second case, the patient’s posttransplant biopsy showed thrombotic microangiopathy with moderate to severe interstitial fibrosis and tubular atrophy. Hematological workup was negative, and the kidney function progressively declined despite a switch to belatacept with low dose cyclosporine from tacrolimus-based regimen.

Among those that did not have PNF, 1 SLK recipient was on dialysis at 1-mo posttransplant (compared with none in the KAL group), and all patients were dialysis free at 3 mo posttransplant.

### Rejection Episodes

Rejection episodes were recorded only if there was biopsy-proven acute rejection in the organ of interest (and not inferred in the nonbiopsied organ based on clinical suspicion). Among SLK recipients, 4 patients (8.1%) underwent kidney biopsies. Only 1 patient (2%) had biopsy-proven borderline rejection whereas the remaining biopsies showed normal findings (n = 1, 2%) and acute tubular injury (n = 2, 4.1%). Nine patients (18%) had liver rejection among SLK recipients. Kidney rejection occurred in 1 KAL recipient (6.7%), while 3 of 34 KAL waitlisted patients (8.8%) experienced liver rejection (Table 2).

### Reasons for Declining KAL Waitlisting via the Safety Net

Among 716 patients alive 60 d post-LTA, only 67 (9.4%) were referred for KAL. Of these, 30 (44.8%) were declined for safety net listing and 3 (4.5%) remained in evaluation. Seventeen (56.7%) were closed after eGFR improved >20 mL/min/1.73 m^2^; the rest (43.3%) were declined for reasons including comorbidity/frailty (2), metastatic cancer (2), nonadherence (3), active smoking/drinking (2), and persistent ascites requiring paracenteses (1). Three patients died during follow-up (2 declined for nonadherence, 1 for smoking/drinking).

### Wait Time for KAL Transplant

**FIGURE 3.**
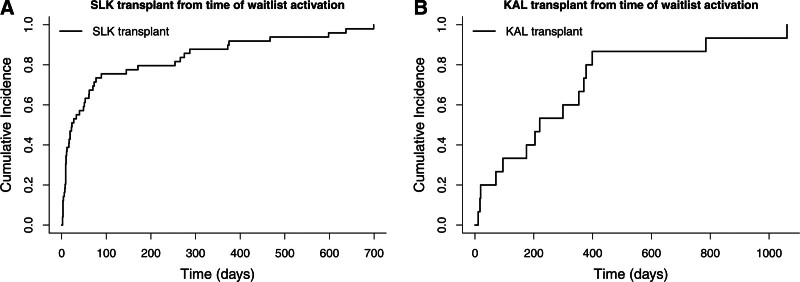
SLK and KAL transplant from the time of waitlist activation. Time zero indicates the time of SLK waitlist activation and kidney waitlist activation in panels (A) and (B), respectively. A, SLK transplant from the time of waitlist activation. B, KAL transplant from the time of waitlist activation. KAL, kidney after liver; SLK, simultaneous liver-kidney.

The median wait time for a KAL transplant after waitlisting was 272 d (IQR, 107–412 d) and after activation was 220 d (IQR, 83–374 d; Figure 3). The median time between the liver transplant and KAL transplant was 448 d (IQR, 325–597 d).

### LTA in Patients Approved for SLK

Three patients initially approved for SLK transplant underwent LTA because of the need for urgent liver transplantation. In 2 cases, this was driven by large blood loss and acute liver decompensation. The third patient, listed for SLK after fulminant liver failure of the first graft, developed severe coagulopathy requiring explantation of the necrotic liver and urgent retransplantation. Post-LTA outcomes varied: the first patient developed recurrent metastatic cholangiocarcinoma with biliary obstruction and died 241 d after transplant while inactive on the KAL waitlist, having been on dialysis for 83 d before and continuing after LTA. The second patient was listed inactive for KAL but later removed as eGFR improved to consistently >20 mL/min/1.73 m^2^. The third patient’s kidney function recovered to 40–50 mL/min/1.73 m^2^ following liver retransplantation, leading to removal from the KAL waitlist.

## DISCUSSION

Our single-center study aims to provide a descriptive and granular overview of our experience after the safety net policy for KAL transplant was introduced on August 10, 2017. We found that (1) mortality among LTA patients within the first 60 d was unrelated to poor kidney function or a delay in kidney transplantation, (2) majority of patients received a KAL transplant within 2 y of liver transplant via the safety net pathway with no mortality at 3 y of follow-up, (3) <10% of liver transplant recipients were ultimately referred and evaluated for a KAL transplant; half of those waitlisted had native kidney recovery leading to delisting, and (4) the kidney allograft function at 1 y was comparable in SLK and KAL recipient despite a disproportionately higher number of low KDPI (0%–20%) kidneys transplanted into SLK versus KAL patients.

Of 34 patients waitlisted, 15 received KAL transplants, 10 within the first year. Most occurred within 2 y of LTA. Prior studies show similar survival for KAL within 60–365 d and SLK recipients, and safety net outcomes comparable to LTA without CKD.^2,18,19^ Our data confirm early LTA deaths and KAL waitlist deaths were not kidney-related. Allocation policy aims to address kidney supply-demand mismatch and avoid SLK for reversible AKI. Many patients were delisted after renal recovery, allowing kidneys to be used for others. For those declined for KAL because of comorbidities, nonadherence, cancer, or substance use, transplant would not have improved outcomes.

One of the strengths of our study is the ability to follow the clinical course of individual patients with detailed chart review. We were able to track the outcomes in patients who were approved for SLK transplant but received LTA. We showed that in this highly selected cohort, LTA with safety net listing (and delayed kidney transplantation) had acceptable outcomes. In fact, although 1 patient died of recurrent cholangiocarcinoma, the other 2 recovered enough kidney function to be delisted from the KAL waitlist. This highlights the lack of accuracy in predicting who actually needs an SLK transplant versus an LTA despite implementing the current eligibility criteria.

Kidney allograft function at 1 y was similar for KAL and SLK, although SLK recipients more often received lower KDPI kidneys. Two SLK patients had PNF; only 1 was recipient-related. That patient was relisted but remained inactive because of hypotension, refractory anemia, and later died after prolonged hospitalization with complications. No KAL transplant had PNF. In addition, 2 SLK recipients died with functioning kidney allografts within the first year of transplant (1 from cholangiocarcinoma and 1 from graft versus host disease) compared with no deaths in the KAL group. Taking the deaths and PNF into account, one could say that 1-y kidney allograft failure rate is higher in SLK compared with KAL recipients, and that a majority of these were not related to donor issues. Interestingly, the MELD scores for these SLK patients were lower. Cullaro et al^20^ have previously looked at pre-policy change United Network for Organ Sharing data and showed that the risk for kidney allograft failure was significantly higher in patients with higher MELD scores (>25) compared with KAL transplant recipients.

A closer look at KAL waitlisted patients did not show a higher risk of eGFR <20 mL/min/1.73 m^2^ with prior proteinuria (60% versus 66.7%; *P* = 0.76) or presence of pre-LTA diabetes (50% versus 33.3%; *P* = 0.46). The lack of statistical significance in our study is likely reflective of our small sample size and will need to be reexamined in a larger study as proteinuria and diabetes are known predictors of poor kidney function. A prior study by Werneburg et al^21^ showed that hepatorenal syndrome as the sole cause of renal disease is a predictor of improvement in native kidney function. An observational study looking at SLK recipients transplanted between 2002 and 2017 at 6 large US transplant centers showed that pretransplant diabetes (hazard ratio [HR], 1.45; 95% confidence interval [CI], 1-2.15), nonalcoholic steatohepatitis (HR, 1.58; 95% CI, 1.01-2.45), and delayed graft function (HR, 1.72; 95% CI, 1.1-2.71) were associated with increased risk of stage 4–5 CKD at a median follow-up time of 64 mo.^5^ Future studies should look at the absence of proteinuria and preexisting diabetes as predictors of native renal recovery in LTA patients with AKI to help provide risk stratification to ESKD and SLK versus safety net listing.

Our study has several limitations. First, because of the limited size of our study population, we were unable to perform meaningful statistical analysis on our results. Therefore, any conclusions we draw will have to be tested in a larger number of patients. Second, we acknowledge the issue of immortal time bias favoring KAL transplant that is inherent in our analysis despite having granular data on these deaths. However, we were able to show that deaths occurred in only a minority of patients within 60 d (N = 9, 1.3%). Additionally, by doing a detailed chart review, we were able to ascertain the clinical events that preceded the deaths in these patients, allowing us to conclude that these deaths were unlikely to be directly attributable to a delay in KAL transplant. Third, there may be selection bias favoring KAL transplants as well given some patients are inevitably declined because of factors such as comorbidity burden and nonadherence among others as seen in our study. Fourth, this is a single-center study, and our center practices may not necessarily translate to practices across the country. For example, we have a tendency to list patients inactive for KAL while following their kidney function and overall patient functionality with the option of delisting if the kidney function improves or patient functionality declines—this may have allowed us to ensure all patients take full advantage of the safety net. Fifth, the kidney pumping data in the forms submitted to Organ Procurement and Transplantation Network by our center does not reflect Organ Procurement Organization practices where the donor kidney came from. It is possible that the donor kidney was pumped by the Organ Procurement Organization but placed on ice before being transferred to our center. At our center, we do not generally place kidneys on a pump when they are obtained on ice as most SLK transplants are done in a nonstaged manner. However, if kidneys are obtained on a pump, they are continued on pump until implantation. Exact duration of kidney pumping was also not available on the electronic health records or the data forms and represents a significant limitation in current data collection practices. At our center, estimated duration for kidney pumping ranges from 2 to 6 h, and there is no standardized protocol differentiating SLK from KAL cases. While pumping may improve immediate graft function and reduce delayed graft function in kidney transplant alone recipients, SLK recipients present unique challenges—such as prolonged surgery, liver failure, and coagulopathy—that likely have a greater impact on early kidney outcomes than pumping practices. Unfortunately, we were unable to do further analysis on the impact of kidney pumping practices as only 1 patient had a transplant with a pumped kidney. We acknowledge that our data will need to be interpreted cautiously by other transplant centers where pumping practices may be more prevalent given the low use of kidney pumping at our center based on available Organ Procurement and Transplantation Network data.

Despite these limitations, our study has 3 distinct strengths. First, we provide granular review of mortality and graft loss to distinguish CKD versus patient-related causes. Second, to our knowledge, this is the first study to report native renal function recovery or nonrecovery following safety net listing and pre-LTA. Third, instead of only looking at patients who were waitlisted or transplanted, we evaluated the entire cohort of patients that were evaluated and approved for a liver transplant with assessment of SLK eligibility or KAL eligibility to describe their clinical course if they were declined for the transplant or never assessed for 1 despite fulfilling eligibility. The granularity in our single-center data supplements the findings of prior national registry studies where detailed clinical course and assessment of eligibility are difficult because of the constraints of available data elements within the registry.

In conclusion, we show that the new allocation policy allowed our center to successfully stratify patients who can receive LTA, potentially allowing kidneys to be transplanted to other patients in need, without causing harm to LTA recipients. We also showed that kidney allograft function in the SLK group was similar to the KAL group despite a markedly higher proportion of low KDPI (0%–20%) kidneys transplanted into SLK recipients, suggesting that KAL is a viable option for carefully selected patients. Our findings are descriptive and exploratory. This will need to be replicated by other centers and on a larger scale in prospective studies to see if they hold true.

## Supplementary Material


