# Supplementary material for: Outcomes of Simultaneous Liver-Kidney Transplant and Kidney After Liver Transplant Using the Safety Net Criteria—A Single-center Experience
Source: Transplant Direct. 2026 Apr 22;12(5):e1940. doi: 10.1097/TXD.0000000000001940 (PMC13105797; doi:10.1097/TXD.0000000000001940)
Supplement: Supplementary file 1 [file txd-12-e1940-s001.pdf]

Table S1. Causes of kidney disease

	SLK transplant		KAL transplant	
		N=49		N=15
Hepatorenal syndrome		15		5
Hepatorenal syndrome + other possible causes	Lupus	1	Rhabdomyolysis	1
	Calcineurin nephrotoxicity	3	Contrast induced nephropathy post transarterial chemoembolization	1
	Calcineurin nephrotoxicity with cryoablation	1	Calcineurin inhibitor nephrotoxicity	2
	Diabetes mellitus	4		
Other causes	Polycystic kidney disease	9	Non-recovery from acute tubular necrosis	3
	Diabetes mellitus	8	AL amyloidosis	1
	Calcineurin nephrotoxicity	3	Diabetes mellitus and calcineurin inhibitor nephrotoxicity	1
	Oxalate nephropathy	2	Calcineurin nephrotoxicity	1
	IgA nephropathy	1		
	Non-recovery from acute tubular necrosis	1		
	C3 glomerulopathy	1		
